# Early Exposure to Traffic-Related Air Pollution, Respiratory Symptoms at 4 Years of Age, and Potential Effect Modification by Parental Allergy, Stressful Family Events, and Sex: A Prospective Follow-up Study of the PARIS Birth Cohort

**DOI:** 10.1289/EHP239

**Published:** 2016-05-24

**Authors:** Fanny Rancière, Nicolas Bougas, Malika Viola, Isabelle Momas

**Affiliations:** 1 Laboratoire Santé Publique et Environnement, EA4064, Faculté de Pharmacie de Paris, Université Paris Descartes, Sorbonne Paris Cité, Paris, France; 2 Cellule Cohorte, Direction de l’Action Sociale de l’Enfance et de la Santé, Mairie de Paris, Paris, France

## Abstract

**Background:**

The relation between traffic-related air pollution (TRAP) exposure and the incidence of asthma/allergy in preschool children has been widely studied, but results remain heterogeneous, possibly due to differences in methodology and susceptibility to TRAP.

**Objectives:**

We aimed to study the relation of early TRAP exposure with the development of respiratory/allergic symptoms and asthma during preschool years, and to investigate parental allergy, “stressful” family events, and sex as possible effect modifiers.

**Methods:**

We examined data of 2,015 children from the PARIS birth cohort followed up with repeated questionnaires completed by parents until age 4 years. TRAP exposure in each child’s first year of life was estimated by nitrogen oxides (NO_x_) air dispersion modeling, taking into account both home and day care locations. Association between TRAP exposure and patterns of wheezing, dry night cough, and rhinitis symptoms was studied using multinomial logistic regression models adjusted for potential confounders. Effect modification by parental history of allergy, stressful family events, and sex was investigated.

**Results:**

An interquartile range (26 μg/m^3^) increase in NO_x_ levels was associated with an increased odds ratio (OR) of persistent wheezing at 4 years (adjusted OR = 1.27; 95% confidence interval: 1.09, 1.47). TRAP exposure was positively associated with persistent wheeze, dry cough, and rhinitis symptoms among children with a parental allergy, those experiencing stressful family events, and boys, but not in children whose parents did not have allergies or experience stressful events, or in girls (all interaction *p*-values < 0.2).

**Conclusions:**

This study supports the hypothesis that not all preschool children are equal regarding TRAP health effects. Parental history of allergy, stressful family events, and male sex may increase their susceptibility to adverse respiratory effects of early TRAP exposure.

**Citation:**

Rancière F, Bougas N, Viola M, Momas I. 2017. Early exposure to traffic-related air pollution, respiratory symptoms at 4 years of age, and potential effect modification by parental allergy, stressful family events, and sex: a prospective follow-up study of the PARIS birth cohort. Environ Health Perspect 125:737–745; http://dx.doi.org/10.1289/EHP239

## Introduction

The prevalence of respiratory and allergic diseases in early childhood has been rising globally, which is unlikely to be attributable to genetic changes only. These multifactorial diseases are associated with both individual and environmental factors. Recent decades have seen a change in air pollution profile in western urban areas, with motor vehicle traffic emissions now as a major source of air pollution (Mayer 1999). Traffic-related air pollution (TRAP) is known to worsen existing respiratory disease (Weinmayr et al. 2010). However, despite substantial literature on the relation between TRAP and the development of asthma and allergy in preschool years, results are still heterogeneous and some uncertainties persist (Bråbäck and Forsberg 2009). For instance, although a meta-analysis of 19 published studies showed evidence for a relationship between TRAP exposure and wheezing in preschool children (Gasana et al. 2012), pooled analyses of five European birth cohorts within the European Study of Cohorts for Air Pollution Effects (ESCAPE) project revealed no significant association of TRAP exposure in early years of life with asthma prevalence at 4–5 years (Mölter et al. 2015) or sensitization to inhalant or food allergens at 4 years (Gruzieva et al. 2014). These inconsistencies in findings may be attributable to methodological issues such as variability in the assessment of TRAP exposure (Brauer 2010) and the definition of health outcomes, and to the possible existence of susceptible subgroups.

In birth cohort studies, TRAP exposure of preschool children has been studied using various indicators: distance to traffic, land-use regression (LUR) models, and, less often, air dispersion models (Bowatte et al. 2015). There are also differences in TRAP pollutants that authors considered [e.g. nitrogen dioxide (NO_2_), nitrogen oxides (NO_x_), particulate matter with an aerodynamic diameter ≤ 10 μm (PM_10_) or ≤ 2.5 μm (PM_2.5_), soot, black carbon], as well as in place and timing of exposure, with most studies only considering the home address at birth, and a few studies considering residential mobility and/or other locations where infants spend time such as day care center. Furthermore, asthma may be difficult to reliably diagnose at preschool age when the clinical symptoms of asthma are variable and nonspecific, and asthma-like or allergy-like symptoms other than wheeze have not been extensively explored.

Besides methodological considerations, another explanation may be related to differences in vulnerability to TRAP. Even if early childhood is a critical period of vulnerability for everyone because of continued development and maturation of the lung and immune system, certain children may be at increased risk for adverse health effects from TRAP (Sacks et al. 2011). In particular, atopy may play a role as an effect modifier, but results from the literature are not entirely consistent. Stronger associations between TRAP exposure and asthma were observed in atopic children in some studies (Dell et al. 2014; Janssen et al. 2003; Schultz et al. 2012) and in nonatopic children in other studies (Gruzieva et al. 2013; McConnell et al. 2006; Nordling et al. 2008). Moreover, emerging research indicates that stress may play a role in increasing the deleterious effect of TRAP on school-age children’s respiratory health (Chen et al. 2008; Clougherty et al. 2007; Islam et al. 2011; Shankardass et al. 2009); but, to our knowledge, no such studies have been conducted in preschool children. Further, whether the susceptibility to the effects of TRAP differs between preschool boys and girls remains unclear. Some authors reported evidence of stronger effects in boys (Gehring et al. 2002) or in girls (Nordling et al. 2008), whereas others did not find any evidence for an effect measure modification by sex (Gruzieva et al. 2014). Last, gene–environment interactions may also partially explain observed heterogeneity in associations between TRAP exposure and the incidence of asthma and allergic outcomes, as suggested by findings from the Traffic, Asthma, and Genetics (TAG) study (Fuertes et al. 2013; MacIntyre et al. 2014).

Consequently, much needed are longitudinal studies with refined assessment of TRAP exposure and insight into factors that may modify the effect of children’s TRAP exposure on respiratory and allergic morbidity. Especially, birth cohort studies are essential to understand the life course and childhood predictors of asthma and allergy, and the complex interplay between heritable and environmental factors (Bousquet et al. 2014).

As part of the Pollution and Asthma Risk: an Infant Study (PARIS) birth cohort, the aims of this study were *a*) to investigate the association between TRAP exposure in early life and the history of respiratory symptoms and asthma during the preschool years, and *b*) to explore whether certain groups of preschool children are more prone to develop respiratory symptoms and asthma in relation to TRAP exposure, focusing on parental allergy, “stressful” family events, and sex.

## Methods

### Study Design and Setting

Data were collected from birth to age 4 years, including a face-to-face interview with the mother at the maternity hospital, a phone interview at 1 month, and regular self-administered questionnaires filled in by parents when their child was 1, 3, 6, 9, 12, and 18 months and 2, 3, and 4 years of age.

### Participants

PARIS is a population-based birth cohort study that enrolled 3,840 newborn babies born between 2003 and 2006 in five Paris maternity hospitals. Information about medical and sociodemographic eligibility criteria and methods of selection was previously published (Clarisse et al. 2007). Briefly, PARIS included single-birth, full-term newborns, without malformation, and with an uncomplicated birth and neonatal period. Exclusion criteria included infants whose mothers were < 18 years of age, did not receive medical care during pregnancy, had alcohol or drug addiction, or had difficulty speaking French. The French Ethics Committee approved the study protocol and written informed consent was obtained from the parents.

### Variables

***Health outcomes.*** Respiratory symptoms suggestive of asthma or allergy were assessed by standardized questions from the International Study of Asthma and Allergies in Childhood (Asher et al. 1995), used in the European consortium MeDALL (Mechanisms of the Development of ALLergy) (Antó et al. 2012; Pinart et al. 2014). At ages 1, 2, 3 and 4 years, parents were asked about the occurrence in the previous year of wheezing (“In the last 12 months, has your child had wheezing or whistling in the chest?”), dry night cough (“In the last 12 months, has your child had a dry cough at night, apart from a cough associated with a cold or chest infection?”), and rhinitis symptoms (“In the last 12 months, has your child had a problem with sneezing, or a runny or blocked nose when he/she did not have a cold or the flu?”).

We hypothesized that the effects of TRAP exposure on respiratory health during preschool years may differ depending on the time of onset and the persistence of the symptoms. To account for the temporality of symptoms during preschool years, children were categorized into four classes according to the trajectory of each of these three symptoms between 0 and 4 years:

No symptom
Early-transient: symptom occurring between 0 and 2 years of age and not persisting later;
Late-onset: symptom occurring between 2 and 4 years of age;
Persistent: symptom occurring between 0 and 2 years of age and persisting later.

Each year, parents were asked whether a doctor had ever diagnosed their child with asthma. Asthma ever at 4 years was defined as asthma ever doctor-diagnosed between 0 and 4 years. Asthma ever with current respiratory symptoms at 4 years was defined as asthma ever in addition to respiratory symptoms (wheezing, dry night cough) in the previous year at 4 years.

***TRAP exposure.*** We used the ExTra index developed by Sacre et al. (1995) to estimate ambient concentrations of traffic-related air pollutants such as nitrogen oxides [NO_x_ = nitrogen monoxide (NO) + NO_2_], taking into account the different places (home and day care) attended by children during their first year of life.

At each follow-up time point, the parents provided in a specific questionnaire the home and day care addresses (including the floor number), as well as the time spent at day care (number of hours per week). We derived the time spent at home as the remaining time. Addresses were geocoded using traditional maps (scale: 1/15,000 or 1/12,500), cadastral maps, and/or the geographic information system (GIS) of the Paris municipality.

Briefly, the ExTra index relies on an air dispersion model adapted from the Danish Operational Street Pollution Model (OSPM) by the French Scientific and Technical Center for Building (CSTB) and the French Institute of Science and Technology for Transport, Development and Networks (IFSTTAR), and has been validated by our research team (Reungoat et al. 2003). Briefly, NO_x_ concentrations measured over 6 weeks with passive samplers were compared with NO_x_ concentrations modeled using the ExTra index, at 100 sites in four French cities including Paris. There were highly significant correlations (*r* = 0.89, *p* < 10^–4^ in Paris) and good intraclass correlation coefficients (*R* = 0.89 in Paris) between the two series of values.

The ExTra index integrates a regional component corresponding to the background NO_x_ levels measured by the Paris air quality monitoring network (AIRPARIF) and a local component modeling NO_x_ levels in front of the different locations attended by children. The modeling step required the preliminary collection of a large amount of data such as topographical features of each relevant location (residence, day care) for each child in the cohort (height of buildings and width of pavements and road collected using a GIS), meteorological data (wind direction and speed supplied by the local weather bureau), and traffic density in the street (resulting from counting or estimating).

The ExTra index is composed of subindices corresponding to the different life periods of each child. A life period is defined as the maximum time period during which no change in locations (home, day care) or time spent in these locations occurred. The ExTra index, expressed in μg/m^3^ NO_2_ equivalent, was then calculated for each child’s first year of life as the weighted average of the different subindices. In summary, the concentration assigned to each child can be expressed as following, for all life periods *i*, with *C* the concentration of NO_x_, *T* the percent of time spent in the place during the period, and *D* the duration of the period in days.


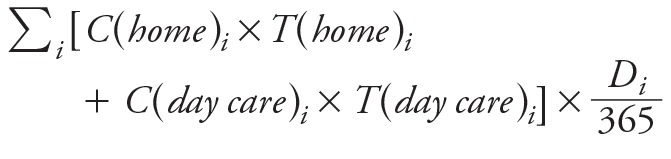


The modeling of TRAP exposure during the fourth year of life taking into account home, day care and school locations is ongoing in our study.

***Family and home characteristics.*** At the maternity hospital, the mother was questioned about family characteristics including presence of older siblings, parental education, occupation, and history of asthma, eczema, and allergic rhinitis, and maternal active and passive smoking during pregnancy. Family socioeconomic status (SES) was based upon the highest position among the two parents and divided into three categories (low/medium/high) as previously described (Rancière et al. 2013b). Parental history of allergy was defined as at least one parent with a history of asthma, eczema, or allergic rhinitis.

When the child was 1 month old, a trained interviewer administered a phone questionnaire to one of the parents about housing characteristics and living habits, and occurrence of “stressful” family events. The self-administered questionnaires mailed to the families between 3 months and 4 years of age produced an update of previously collected data and documented duration of breastfeeding and type of day care setting. We considered a child to be exposed to “stressful” family events if the parents reported a separation/divorce, a loss of job, a serious health problem (e.g., chronic disease, cancer, depression, surgery, hospitalization) in any family member or close relative or the death of a family member or a close relative, during the first 2 years of life.

### Statistical Methods

The main characteristics of PARIS children, whether or not included in the present study, were analyzed using chi-squared tests or Student’s *t*-tests.

The association of early-life TRAP exposure with asthma and patterns of respiratory symptoms was assessed using multinomial logistic regression (for the wheeze, dry cough, and rhinitis outcomes) or logistic regression (for the dichotomous asthma outcomes) adjusted for the relevant variables. Covariates were selected for inclusion in the statistical models using a directed acyclic graph (DAG) built using DAGitty version 2.2 (Textor et al. 2011). The DAG is presented in Figure S1. Relationships between each of the variables were assigned based on knowledge of the literature regarding these associations. Given the assumptions described in the DAG theory, we identified the minimal sufficient set of adjustment variables for estimating the direct effect of TRAP exposure on respiratory health. Covariates selected for inclusion in the multivariable models were sex, birth weight (continuous), family SES (low, medium, high), maternal education (high school education or less, at least some college), exclusive breastfeeding during the first 3 months (no, yes), type of day care during the first 6 months (no day care, at home, at a childminder’s home, in a day care center), maternal smoking during pregnancy (no, yes), exposure to environmental tobacco smoke at home during the first year (no, yes), body mass index ≥ 85th percentile for age and sex at 2–3 years (no, yes), visible mold in the home at birth (no, yes), gas for cooking/heating in the home at birth (no, yes), and stressful family events (no, yes). Given our research question, models were also adjusted for maternal and paternal history of allergy (no, yes), which did not result in any biasing path.

Children with complete data for all covariates were included in the final multivariable models. Results were expressed as adjusted odds ratios (OR) and their 95% confidence intervals (CI). TRAP exposure levels were entered as a continuous variable, and results are presented for an interquartile range (IQR) increase in NO_x_ levels within the PARIS birth cohort (26 μg/m^3^ NO_2_ equivalent).

Possible effect measure modification by parental history of allergy (based on either parent, or based on the mother only, father only, or both), stressful family events, and sex was explored by testing multiplicative interactions using an alpha of 0.2.

In a subsample of about 800 children for whom TRAP exposure during the fourth year of life has already been modeled, we performed a sensitivity analysis including TRAP exposure levels during both the first year (early exposure) and the fourth year (later exposure).

All analyses were performed using Stata/SE version 13.1 (StataCorp).

## Results

### Participants

Results are given for 2,015 children for whom information about TRAP exposure level during the first year and natural history of at least one respiratory symptom during the first 4 years were available. Twenty-eight percent of the children not included had to stop the follow-up before their 4th birthday because they moved outside the study area, a withdrawal criterion specified in the protocol (Clarisse et al. 2007). The main characteristics of children included in this study are presented in Table 1. Compared with children from the PARIS birth cohort not included, the 2,015 children included in this study were more likely to live in a family with a high SES and less likely to have been exposed to maternal smoking during pregnancy. Parents of children included in the study more often reported a paternal history of asthma, eczema, or allergic rhinitis.

**Table 1:** Baseline characteristics of children from the PARIS cohort included (*n* = 2,015) and not included (*n* = 1,825) in the present study.

Baseline characteristics	Included	Not included	*p*-Value
Male sex	1,032 (51.2)	941 (51.6)	0.83
Birth weight (kg)	3.40 ± 0.39	3.40 ± 0.40	0.77
Family socioeconomic status			< 0.001
Low	122 (6.0)	244 (13.4)
Medium	525 (26.1)	555 (30.4)
High	1,368 (67.9)	1,026 (56.2)
Place of residence at birth			0.98
Paris city	1,277 (63.4)	1,156 (63.3)
Paris suburbs	738 (36.6)	669 (36.7)
Older siblings	885 (43.9)	776 (42.5)	0.38
Maternal history of asthma, eczema, or allergic rhinitis	759 (37.7)	651 (35.7)	0.20
Paternal history of asthma, eczema, or allergic rhinitis	745 (37.1)	603 (32.2)	0.01
Maternal use of antibiotics during pregnancy	296 (14.7)	261 (14.3)	0.73
Maternal smoking during pregnancy	203 (10.1)	230 (12.6)	0.01
Cat allowed in the child’s bedroom just after birth	160 (8.0)	124 (7.2)	0.36
Visible mold in the home just after birth	312 (15.5)	284 (16.4)	0.46
Use of gas for cooking or heating in the home just after birth	1,096 (55.5)	960 (57.2)	0.28
Data are shown as *n* (%) or mean ± SD. Total numbers may not be equal to 2,015 and 1,825 for some characteristics due to missing data.			

### Levels of TRAP Exposure

Levels of early exposure to NO_x_ ranged from 39 to 257 μg/m^3^ NO_2_ equivalent, with a median (IQR) of 75 (66–89) μg/m^3^. Children exposed to NO_x_ levels above the median level were more likely to be from families with a high SES, to have been exposed to maternal smoking during pregnancy, and to live in a home with gas used for heating and/or cooking (Table 2). TRAP exposure levels were significantly higher in the inner city than in the suburbs, with a median (IQR) of 77 (69–91) μg/m^3^ and 68 (58–86) μg/m^3^ NO_2_ equivalent, respectively (*p* = 0.008).

**Table 2:** Characteristics of children from the PARIS cohort included in the study according to median level of traffic-related air pollution exposure during the first year (*n* = 2,015).

Characteristics^*a*^	NO_x _< 75 μg/m^3^ (*n* = 973)	NO_x _≥ 75 μg/m^3^ (*n* = 1,042)	*p*-Value
Sex			0.84
Male	496 (51.0)	536 (51.4)
Female	477 (49.0)	506 (48.6)
Birth weight	3.42 ± 0.39	3.39 ± 0.40	0.12
Family socioeconomic status			0.01
Low	69 (7.1)	53 (5.1)
Medium	274 (28.2)	251 (24.1)
High	630 (64.7)	738 (70.8)
Maternal education			0.58
High school education or less	109 (11.2)	109 (10.5)
At least some college	862 (88.8)	933 (89.5)
Missing (*n*)	2	0
Maternal history of asthma, eczema, or allergic rhinitis			0.56
No	600 (61.7)	655 (62.9)
Yes	373 (38.3)	386 (37.1)
Missing (*n*)	0	1
Paternal history of asthma, eczema, or allergic rhinitis			0.72
No	606 (62.5)	656 (63.3)
Yes	364 (37.5)	381 (36.7)
Missing (*n*)	3	5
Maternal smoking during pregnancy			0.04
No	889 (91.4)	923 (88.6)
Yes	84 (8.6)	119 (11.4)
Visible mold in the home			0.92
No	822 (84.6)	877 (84.4)
Yes	150 (15.4)	162 (15.6)
Missing (*n*)	1	3
Use of gas for cooking or heating in the home			0.003
No	455 (48.0)	425 (41.3)
Yes	493 (52.0)	603 (58.7)
Missing (*n*)	25	14
Exclusive breastfeeding during the first 3 months			0.65
No	678 (70.2)	736 (71.1)
Yes	288 (29.8)	299 (28.9)
Missing (*n*)	7	7
Day care during the first 6 months			0.87
No	374 (39.8)	379 (38.4)
Yes, at home	158 (16.8)	174 (17.6)
Yes, at a childminder’s home	208 (22.1)	229 (23.2)
Yes, in a day care center	200 (21.3)	206 (20.8)
Yes, but type not specified (*n*)	33	54
Exposure to smoking at home during the first year			0.13
No	717 (75.3)	735 (72.3)
Yes	235 (24.7)	282 (27.7)
Missing (*n*)	21	25
Stressful family events during the first 2 years^*b*^			0.51
No	521 (54.8)	540 (53.3)
Yes	430 (45.2)	473 (46.7)
Missing (*n*)	22	29
Body mass index ≥ 85th percentile for age and sex at 2–3 years			0.06
No	755 (79.5)	770 (75.9)
Yes	195 (20.5)	245 (24.1)
Missing (*n*)	23	27
Data are shown as *n* (%) or mean ± SD. ^***a***^Characteristics are at birth unless otherwise specified. ^***b***^Among parental separation/divorce, parental loss of job, serious health problem, or death of a family member or close relative.			

### Main Results

Overall, early TRAP exposure was significantly associated with persistent wheeze at age 4 years (OR = 1.27; 95% CI: 1.09, 1.47) compared with children without wheeze during the first 4 years of life (Table 3). No such associations were observed for early-transient and late-onset wheeze. In addition, TRAP exposure was significantly associated with increased OR for asthma ever (OR = 1.15; 95% CI: 1.01, 1.31) and asthma ever with current respiratory symptoms at 4 years (OR = 1.20; 95% CI: 1.02, 1.41). There was no significant association of TRAP exposure with any patterns of dry night cough or rhinitis symptoms.

**Table 3:** Association between traffic-related air pollution exposure during the first year and respiratory health during preschool years in the PARIS cohort.

Respiratory health during the first 4 years of life	*n* (%)	aOR (95% CI)	*p*-Value
Wheeze
No (reference)	1,181 (69.1)	1
Early-transient	317 (18.5)	1.03 (0.91, 1.17)	0.67
Late-onset	86 (5.0)	1.09 (0.89, 1.33)	0.40
Persistent	126 (7.4)	1.27 (1.09, 1.47)	0.002
Dry night cough
No (reference)	1,032 (60.6)	1
Early-transient	190 (11.2)	1.01 (0.87, 1.18)	0.87
Late-onset	265 (15.5)	1.03 (0.91, 1.18)	0.63
Persistent	217 (12.7)	1.11 (0.97, 1.27)	0.13
Rhinitis symptoms
No (reference)	934 (55.3)	1
Early-transient	300 (17.7)	0.95 (0.83, 1.09)	0.45
Late-onset	175 (10.4)	1.06 (0.91, 1.24)	0.45
Persistent	281 (16.6)	1.09 (0.96, 1.24)	0.18
Asthma ever at 4 years
No (reference)	1,517 (87.2)	1
Yes	223 (12.8)	1.15 (1.01, 1.31)	0.03
Asthma ever with current respiratory symptoms at 4 years
No (reference)	1,595 (93.1)	1
Yes	119 (6.9)	1.20 (1.02, 1.41)	0.03
Notes: aOR, adjusted odds ratio; CI, confidence interval. Odds ratios are calculated for an interquartile range (26 μg/m^3^ NO_2_ equivalent) increase in average NO_x_ levels during the first year of life. The categorical outcomes were modeled using multinomial logistic regression models. Models were adjusted for sex, birth weight, family socioeconomic status, maternal education level, maternal history of asthma, allergic rhinitis, or eczema, paternal history of asthma, allergic rhinitis, or eczema, maternal smoking during pregnancy, exposure to environmental tobacco smoke at home during the first year, exclusive breastfeeding during the first 3 months, type of child care during the first 6 months, stressful family events during the first 2 years, body mass index ≥ 85th percentile for age and sex at 2–3 years, use of gas for cooking or heating in the home, and visible mold in the home.			

**Figure 1:**
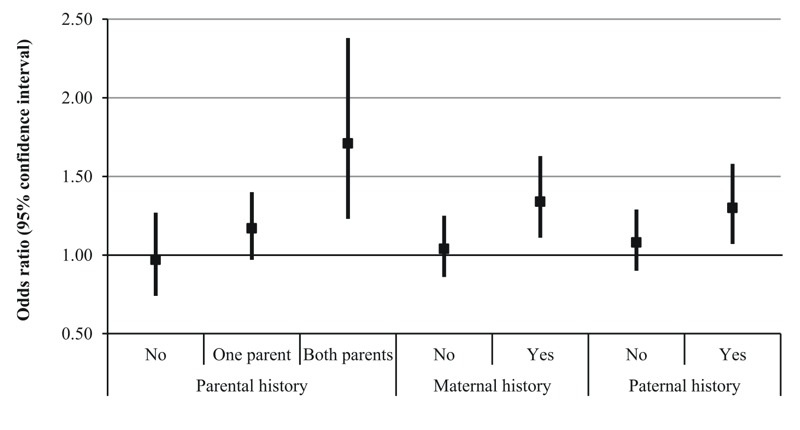
Association between traffic-related air pollution exposure and asthma ever at 4 years according to several definitions of parental history of allergy in the PARIS cohort. Odds ratios were calculated for an interquartile range (26 μg/m^3^ NO_2_ equivalent) increase in average NO_x_ levels during the first year of life. Models were adjusted for the same variables as in Table 3, except maternal and paternal history of allergy for the parental history models, maternal history of allergy for the maternal history models, and paternal history of allergy for the paternal history models.

Associations of TRAP exposure with persistent respiratory symptoms appeared to be modified by parental history of allergy and stressful family events (Table 4). TRAP exposure was positively associated with all persistent respiratory symptoms, asthma ever, and asthma ever with current respiratory symptoms in children whose parents reported a history of allergy, but not in children whose parents did not have a history of allergy (all interaction *p*-values ≤ 0.15). Associations also were positive for the same outcomes among children with a history of stressful family events, but not among children without a history of stressful events, though interactions were not significant (interaction *p* > 0.2) for the two asthma outcomes. The highest ORs were observed for persistent wheeze. Furthermore, we explored whether maternal and paternal allergy had different implications for the risk of asthma ever in relation with TRAP exposure, and they did not appear to have differential effects (Figure 1). TRAP exposure was positively associated with asthma ever in children with allergy in one or both parents, but not in children without parental allergy (*p* for interaction = 0.12). The association between TRAP exposure and asthma ever appeared stronger when both parents had a history of allergy (OR = 1.71; 95% CI: 1.23, 2.38) than when only one parent had a history of allergy (OR = 1.17; 95% CI: 0.97, 1.40).

**Table 4:** Association between traffic-related air pollution exposure and respiratory health according to parental history of allergy and stressful family events in the PARIS cohort.

Respiratory health during the first 4 years of life	Parental history of allergy^*a*^					Stressful family events^*b*^				
	No		Yes		*p* for interaction	No		Yes		*p* for interaction
	*n*	aOR (95% CI)	*n*	aOR (95% CI)		*n*	aOR (95% CI)	*n*	aOR (95% CI)	
Wheeze
No (reference)	507	1	677	1		659	1	522	1
Early-transient	112	0.99 (0.80, 1.23)	205	1.06 (0.90, 1.25)	0.64	162	1.01 (0.85, 1.20)	155	1.02 (0.85, 1.24)	0.98
Late-onset	35	0.96 (0.69, 1.34)	51	1.16 (0.90, 1.50)	0.35	46	0.94 (0.68, 1.30)	40	1.26 (0.97, 1.63)	0.19
Persistent	44	1.05 (0.78, 1.41)	82	1.42 (1.18, 1.71)***	0.12	62	1.06 (0.82, 1.37)	64	1.46 (1.19, 1.79)***	0.08
Dry night cough
No (reference)	458	1	576	1		574	1	458	1
Early-transient	72	1.00 (0.78, 1.28)	118	1.04 (0.85, 1.27)	0.81	99	0.99 (0.79, 1.23)	91	1.05 (0.84, 1.31)	0.68
Late-onset	96	0.89 (0.71, 1.12)	170	1.13 (0.96, 1.32)	0.10	155	1.07 (0.90, 1.27)	110	1.00 (0.81, 1.23)	0.52
Persistent	63	0.85 (0.64, 1.15)	154	1.22 (1.04, 1.44)**	0.03	102	0.97 (0.78, 1.20)	115	1.29 (1.08, 1.55)**	0.09
Rhinitis symptoms
No (reference)	415	1	520	1		532	1	402	1
Early-transient	113	0.94 (0.76, 1.15)	188	0.95 (0.79, 1.14)	0.98	161	0.96 (0.80, 1.15)	139	0.97 (0.79, 1.19)	0.91
Late-onset	74	0.98 (0.78, 1.25)	101	1.11 (0.91, 1.36)	0.41	91	1.06 (0.85, 1.31)	84	1.06 (0.85, 1.33)	0.91
Persistent	78	0.79 (0.59, 1.04)	203	1.21 (1.04, 1.40)**	0.01	134	0.95 (0.77, 1.16)	147	1.26 (1.06, 1.49)**	0.04
Asthma ever at 4 years
No (reference)	642	1	878	1		828	1	689	1
Yes	63	0.97 (0.74, 1.27)	160	1.23 (1.06, 1.43)**	0.15	117	1.06 (0.88, 1.29)	106	1.25 (1.05, 1.49)**	0.26
Asthma ever with current symptoms at 4 years
No (reference)	668	1	930	1		862	1	733	1
Yes	27	0.76 (0.47, 1.25)	92	1.32 (1.11, 1.58)**	0.04	72	1.09 (0.86, 1.38)	47	1.34 (1.07, 1.68)**	0.29
Notes: aOR, adjusted odds ratio; CI, confidence interval. Odds ratios are calculated for an interquartile range (26 μg/m^3^ NO_2_ equivalent) increase in average NO_x_ levels during the first year of life. The categorical outcomes were modeled using multinomial logistic regression models. Models were adjusted for the same variables as in Table 3 (except for the stratification variables). ^***a***^Among asthma, allergic rhinitis, and eczema in the mother and/or the father. ^***b***^Among parental separation or divorce, parental loss of job, serious health problem, or death of a family member or close relative during the child’s first 2 years of life. **p ≤ 0.01. ***p ≤ 0.001.										

Associations also differed by sex regarding persistent respiratory symptoms and asthma (Table 5). TRAP exposure was significantly associated with persistent wheeze (OR = 1.39; 95% CI: 1.15, 1.69), persistent dry night cough (OR = 1.21; 95% CI: 1.01, 1.45), and persistent rhinitis symptoms (OR = 1.21; 95% CI: 1.03, 1.43) among boys but not girls (all interaction *p* ≤ 0.12). The association with asthma ever was also significant in boys (OR = 1.22; 95% CI: 1.05, 1.43) but not in girls (OR = 1.04; 95% CI: 0.83, 1.32), even though the interaction was not significant (interaction *p* > 0.20). Moreover, TRAP exposure was positively associated with early-transient wheeze in boys but not in girls, and late-onset wheeze in girls but not in boys (interaction *p*-values = 0.09), although the ORs were not significant.

**Table 5:** Association between traffic-related air pollution exposure and respiratory health according to the child’s sex in the PARIS cohort.

Respiratory health during the first 4 years of life	Sex				
	Boys		Girls		*p* for interaction
	*n*	aOR (95% CI)	*n*	aOR (95% CI)	
Wheeze
No (reference)	564	1	617	1
Early-transient	180	1.12 (0.95, 1.32)	137	0.87 (0.71, 1.08)	0.09
Late-onset	49	0.92 (0.66, 1.28)	37	1.24 (0.96, 1.62)	0.09
Persistent	77	1.39 (1.15, 1.69)***	49	1.09 (0.82, 1.44)	0.12
Dry night cough
No (reference)	506	1	526	1
Early-transient	104	1.02 (0.83, 1.26)	86	0.97 (0.76, 1.23)	0.79
Late-onset	154	1.04 (0.87, 1.24)	111	1.02 (0.84, 1.24)	0.94
Persistent	104	1.21 (1.01, 1.45)*	113	0.99 (0.80, 1.23)	0.12
Rhinitis symptoms
No (reference)	450	1	484	1
Early-transient	162	0.92 (0.75, 1.11)	138	0.98 (0.81, 1.19)	0.57
Late-onset	101	1.05 (0.85, 1.30)	74	1.08 (0.85, 1.35)	0.87
Persistent	146	1.21 (1.03, 1.43)*	135	0.93 (0.76, 1.14)	0.06
Asthma ever at 4 years
No (reference)	745	1	772	1
Yes	147	1.22 (1.05, 1.43)**	76	1.04 (0.83, 1.32)	0.26
Asthma ever with current symptoms at 4 years
No (reference)	798	1	797	1
Yes	76	1.25 (1.02, 1.53)*	43	1.12 (0.85, 1.49)	0.53
Notes: aOR, adjusted odds ratio; CI, confidence interval. Odds ratios are calculated for an interquartile range (26 μg/m^3^ NO_2_ equivalent) increase in average NO_x_ levels during the first year of life. The categorical outcomes were modeled using multinomial logistic regression models. Models were adjusted for the same variables as in Table 3, except for sex. *p ≤ 0.05. **p ≤ 0.01. ***p ≤ 0.001.					

Preliminary results on a subgroup of the cohort (*n* = 768) showed that TRAP exposure levels during the first and fourth years were correlated with a correlation coefficient of 0.64 (*p* < 10^–5^). Early TRAP exposure was still positively associated with persistent wheezing when later TRAP exposure was further included in the model (OR = 1.22; 95% CI: 0.87, 1.72 compared with OR = 1.27; 95% CI: 1.00, 1.62 when later exposure was not included), although the OR estimate was no longer statistically significant. This additional adjustment did not result in improvement in model fit (*p* from likelihood-ratio test = 0.74). The OR estimate associated with later TRAP exposure was closer to 1 and was not statistically significant (OR = 1.09; 95% CI: 0.66, 1.79).

## Discussion

### Key Results

In the PARIS prospective birth cohort study, we aimed to explore the association of early-childhood TRAP exposure with the time course of respiratory/allergic symptoms in the first 4 years and asthma ever at 4 years. In the whole study population, TRAP exposure was associated with asthma and persistent wheezing at 4 years of age. We were also interested in determining whether certain groups of preschool children are more likely to be affected by TRAP exposure, and we found evidence for effect modification by parental allergy, occurrence of stressful family events, and sex. Our analyses suggest that adverse respiratory effects of TRAP exposure may be evident only in preschool children with parental history of allergy, in those who experienced stressful family events, or in boys.

### Strengths and Limitations

A major strength of our study is the TRAP exposure assessment. Early TRAP exposure was finely modeled using an air dispersion model. Modeling NO_x_ levels through the ExTra index showed excellent performance in the study of validation (Reungoat et al. 2003). In the literature, these types of models were relatively little used compared to LUR models due to a complicated and time-consuming implementation. Few cohort studies have estimated children’s TRAP exposure by dispersion model: BAMSE (Children, Allergy, Milieu, Stockholm, Epidemiology) in Sweden [NO_x_ and PM_10_ (Nordling et al. 2008)], Oslo birth cohort in Norway [NO_2_ (Oftedal et al. 2009)], Generation R in the Netherlands [NO_2_ and PM_10_ (Sonnenschein-van der Voort et al. 2012)], and the Children’s Health Study in California [NO_x_ (McConnell et al. 2010)]. Only the BAMSE and PARIS cohorts have studied the impact of TRAP exposure on the respiratory health of preschool children.

Another important strength is the inclusion of all the different places in which each child spent time during his/her first year of life as it should reduce misclassification in TRAP exposure, contrary to other studies where only the home address at birth was considered to assess the TRAP exposure during the first year (Brauer et al. 2007; Morgenstern et al. 2007). In older children, McConnell et al. (2010) showed that TRAP exposure at home and school may both contribute to the development of asthma. Results from the Canadian Healthy Infant Longitudinal Development (CHILD) study suggest that accounting for day care to assess early TRAP exposure might reduce exposure misclassification because participants for whom exposure misclassification was less likely (i.e., those spending more than the city median time at home and those who did not attend day care) had stronger associations between exposure to NO_2_ at home and allergic sensitization at age 1 year (Sbihi et al. 2015). However, in preschool children, TRAP exposure at day care has been rarely considered. The reason for this can be cultural, as in the BAMSE birth cohort in Sweden, where only 1% of the children in the study started day care before 12 months of age (Nordling et al. 2008), compared with about two-third in our study.

Moreover, the NO_x_ levels were predicted at the front windows of each home and day care place and not at the ground level, as in LUR models, resulting in a more precise estimation of the actual level of exposure. In birth cohort studies, nitrogen oxides were mostly reported in the form of NO_2_ and more rarely as both NO_2_ and NO (Bowatte et al. 2015), although gas exhaust from traffic contains NO_x_ composed primarily of NO. Another strength of our study is the adjustment of statistical models for variables related to other sources of children’s exposure to NO_x_ (i.e., use of gas and smoking in the home).

Regarding windows of TRAP exposure, we focused on early childhood, a period of high susceptibility due to the immaturity of the lung and immune system (Dietert et al. 2000). In the literature, the impact of TRAP on the development of asthma and allergy appears stronger for exposure during the first year of life than for exposure later, and TRAP exposure occurring in early life has been linked to long-term respiratory manifestations (Gruzieva et al. 2012; Schultz et al. 2012). We present data on TRAP exposure during the first year, before possible asthma diagnosis. As already highlighted in the BAMSE cohort at the same age of 4 years (Melén et al. 2008; Nordling et al. 2008), considering exposure during the first year of life only is a way to minimize possible reverse causality induced by avoidance behavior due to the child’s disease. However, we cannot state with certainty that exposure during the first year alone is responsible for the observed associations, and later exposure between 1 and 4 years may also contribute to the associations observed in our study. In a subgroup of 769 children from the PARIS cohort, we performed a preliminary model including TRAP exposure levels during two time windows (first and fourth years). After adjusting for later TRAP exposure, early TRAP exposure was still positively associated with persistent wheezing, even though our sample size was most probably too small to have sufficient statistical power to detect a significant association.

One limitation of our study is participation. We observed a substantial attrition rate during follow-up. As previously described (Rancière et al. 2013a), this is partly attributable to families moving outside the study area, consistent with the high residential mobility rates observed in Paris. Previous studies found that associations between TRAP and respiratory diseases and asthma were stronger among individuals of lower socioeconomic status (Burra et al. 2009; Wheeler and Ben-Shlomo 2005). Children enrolled in the PARIS cohort were mainly of high SES, and we were unable to study the interaction between TRAP exposure and social deprivation. However, as observed in the BAMSE cohort, children from families of high SES had the highest exposure levels because they live mainly in the inner city where air pollution levels are higher (Nordling et al. 2008). This trend is reversed in the United States where children highly exposed to TRAP tend to be from disadvantaged backgrounds (Gunier et al. 2003). PARIS children living in homes with gas appliances were most highly exposed to TRAP, probably because the old buildings with gas appliances are mostly located in the inner city.

Another limitation is the parental report of symptoms and stressful family events, in common with most large epidemiological studies. We cannot rule out the possibility that parents of symptomatic children are more prone to recall and disclose stressful events, and differential misclassification to occur. However, the events considered are severe life events (e.g., death, divorce/separation, loss of job) that one would expect to be remembered reliably by all parents.

Our main findings in the overall population are consistent with those of other authors. In a large Canadian nested case–control study (*n* = 37,401), Clark et al. (2010) observed a statistically significantly increased risk of asthma diagnosis by age 4 years with increased early-life exposure to traffic-related air pollutants such as NO and NO_2_. In the Prevention and Incidence of Asthma and Mite Allergy (PIAMA) birth cohort study in the Netherlands, Brauer et al. (2007) found a significant positive association between TRAP exposure modeled using LUR at birth address and doctor-diagnosed asthma ever at 4 years (*n* = 2,575). However, contrary to our study, they also found a significant positive association with early wheeze and no significant association with persistent wheeze at 4 years. By contrast, and more in line with our study, exposure to traffic-NO_x_ during the first year of life, modeled using an air dispersion model, was associated with an excess risk of persistent wheezing at 4 years of age in the BAMSE cohort (*n* = 3,515) (Nordling et al. 2008). Other birth cohort studies have studied preschool respiratory symptoms in relation with TRAP exposure, without specifically studying the time course of symptoms, and the results are mixed (Bowatte et al. 2015). For instance, within the Manchester Asthma and Allergy Study (MAAS), Mölter et al. (2014) found no evidence of a significant association between early exposure to PM_10_ and NO_2_ and the prevalence of either asthma or wheeze up to school age (*n* = 1,185).

In addition to variability in exposure assessment and outcomes definitions, another emerging explanation of the mixed results is that certain subgroups of children may be more susceptible to the adverse health effects from TRAP. Our findings suggest that the relationship of early TRAP exposure with asthma and all persistent respiratory symptoms may be modified by parental history of allergy, stressful family events, and sex.

In birth cohort studies, effect modification by parental allergy has been little studied in preschool children. In the BAMSE cohort, children with parents reporting an allergic disease tended to have a stronger association between TRAP and sensitization at 4 years than if the parents did not report any allergic disease, but no differences were observed for asthma, patterns of wheeze, or peak expiratory flow outcomes at 4 years (Nordling et al. 2008). At school age, recent studies argue in favor of a stronger effect of TRAP exposure on atopic children, atopy being defined by IgE sensitization to common allergens (Schultz et al. 2012) or using surrogates such as allergic comorbidity (Dell et al. 2014). The hypothesis of a stronger effect of TRAP in atopic children than in nonatopic children is in line with previous experimental studies, and several hypotheses can be proposed. First, TRAP has been suggested to interact with aeroallergens and might thereby favor allergenic sensitization (Knox et al. 1997; Motta et al. 2006). Second, TRAP has been shown to increase airway inflammation and/or oxidative stress in urban youth, whether asthmatic or not (Patel et al. 2013). Consequently, TRAP may potentiate airway inflammation in already sensitized children and may explain why the effects of TRAP would be more obvious in children predisposed to allergy, as in our study.

There is growing evidence linking psychosocial factors to the development of asthma and allergy (Wright 2005, 2008). Recent studies suggest that stress is associated with an increased susceptibility of children to effects of TRAP exposure on the development of asthma and allergy. A synergistic effect is biologically plausible because stress can affect the immune response and may potentiate the effects of TRAP due to similar physiological mechanisms involving inflammation and oxidative stress (Wright 2011). A few epidemiological studies conducted in school-age children showed that those highly exposed to both air pollution and stress were more likely to suffer from respiratory symptoms such as wheezing and cough (Chen et al. 2008), to be diagnosed with asthma (Clougherty et al. 2007; Shankardass et al. 2009), or to have impaired lung function (Chen et al. 2008; Islam et al. 2011). To our knowledge, no study has examined whether psychosocial factors could also enhance the detrimental effect of TRAP in preschool children. In the PARIS cohort, the stress perceived by parents was not objectively measured, but we hypothesized that the occurrence of adverse events such as parental separation or divorce, parental loss of job, serious health problem, or death of a family member or close relative may cause stress in the affected parents. Even though infants and toddlers may not understand the meaning of these events, there is evidence that they can be affected by parental stress (Wright et al. 2002).

Several epidemiological studies have suggested that TRAP exposure may be differently associated with respiratory symptoms depending on the child’s sex, but the results are not consistent. In Sweden, Nordling et al. (2008) found that persistent wheezing at 4 years was associated with traffic-NO_x_ in girls but not in boys, whereas in our study, TRAP was positively associated with persistent wheezing at 4 years in boys, but not girls. Our findings are consistent with those from a meta-analysis across five birth cohorts within ESCAPE, where exposure to PM_2.5_ at birth address was positively associated with asthma prevalence at age 4–5 years among boys (OR = 1.80; 95% CI: 1.05, 3.09) but not girls (OR = 0.90; 95% CI: 0.47, 1.70) (Mölter et al. 2015). Such sex differences are biologically plausible due to disparities between male and female lung development from the prenatal period through the first years of life (Becklake and Kauffmann 1999), such as earlier appearance of surfactant in female neonatal lungs, and narrower airways in young males than in young females (Carey et al. 2007).

## Conclusions

In summary, our results suggest that stressful family events, parental history of allergy, and sex may increase the susceptibility of preschool children to the respiratory effect of early-childhood TRAP exposure as associated with asthma onset and persistence of respiratory symptoms suggestive of asthma or allergy. These results need confirmation in future birth cohort studies. More research focusing on factors that may modify the effects of TRAP on the development of asthma and allergy onset is needed and will likely prove useful in clarifying these complex relationships.

## Supplemental Material

(374 KB) PDF
