# Supplementary material for: Early Exposure to Traffic-Related Air Pollution, Respiratory Symptoms at 4 Years of Age, and Potential Effect Modification by Parental Allergy, Stressful Family Events, and Sex: A Prospective Follow-up Study of the PARIS Birth Cohort
Source: Environ Health Perspect. 2016 May 24;125(4):737–45. doi: 10.1289/EHP239 (PMC5381976; doi:10.1289/EHP239)
Supplement: (374 KB) PDF [file EHP239.s001.acco.pdf]

Environ Health Perspect
DOI: 10.1289/EHP239

Note to readers with disabilities: *EHP* strives to ensure that all journal content is accessible to all readers. However, some figures and Supplemental Material published in *EHP* articles may not conform to 508 standards due to the complexity of the information being presented. If you need assistance accessing journal content, please contact ehp508@niehs.nih.gov. Our staff will work with you to assess and meet your accessibility needs within 3 working days.

Supplemental Material

Early Exposure to Traffic-Related Air Pollution, Respiratory Symptoms at 4 Years of Age, and Potential Effect Modification by Parental Allergy, Stressful Family Events, and Sex: A Prospective Follow-up Study of the PARIS Birth Cohort

Fanny Rancière, Nicolas Bougas, Malika Viola, and Isabelle Momas

Table of Contents

Figure S1: Directed acyclic graph for estimating the direct effect of early traffic-related air pollution exposure on respiratory health up to 4 years.

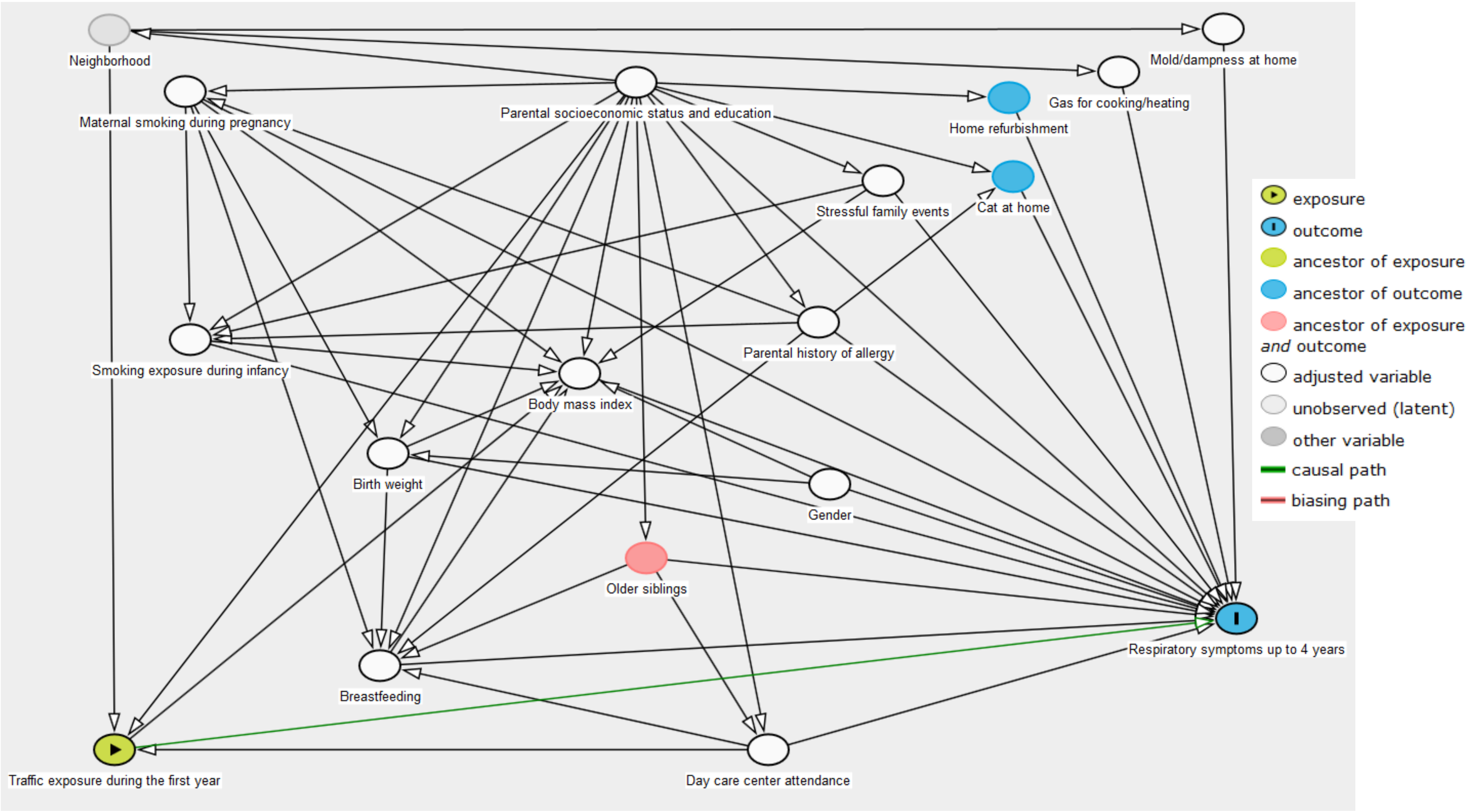

Figure S1: Directed acyclic graph for estimating the direct effect of early traffic-related air pollution exposure on respiratory health up to 4 years.
